# Gene Screening in High-Throughput Right-Censored Lung Cancer Data

**DOI:** 10.3390/onco2040017

**Published:** 2022-10-17

**Authors:** Chenlu Ke, Dipankar Bandyopadhyay, Mario Acunzo, Robert Winn

**Affiliations:** 1 Department of Statistical Sciences and Operations Research, Virginia Commonwealth University, Richmond, VA 23284, USA; 2 Department of Biostatistics, Virginia Commonwealth University, Richmond, VA 23284, USA; 3 Department of Internal Medicine, Virginia Commonwealth University, Richmond, VA 23284, USA; 4 Massey Cancer Center, Virginia Commonwealth University, Richmond, VA 23284, USA

**Keywords:** high dimensional data, lung cancer, right-censored, survival, sure independence screening, TCGA

## Abstract

**Background:**

Advances in sequencing technologies have allowed collection of massive genome-wide information that substantially advances lung cancer diagnosis and prognosis. Identifying influential markers for clinical endpoints of interest has been an indispensable and critical component of the statistical analysis pipeline. However, classical variable selection methods are not feasible or reliable for high-throughput genetic data. Our objective is to propose a model-free gene screening procedure for high-throughput right-censored data, and to develop a predictive gene signature for lung squamous cell carcinoma (LUSC) with the proposed procedure.

**Methods:**

A gene screening procedure was developed based on a recently proposed independence measure. The Cancer Genome Atlas (TCGA) data on LUSC was then studied. The screening procedure was conducted to narrow down the set of influential genes to 378 candidates. A penalized Cox model was then fitted to the reduced set, which further identified a 6-gene signature for LUSC prognosis. The 6-gene signature was validated on datasets from the Gene Expression Omnibus.

**Results:**

Both model-fitting and validation results reveal that our method selected influential genes that lead to biologically sensible findings as well as better predictive performance, compared to existing alternatives. According to our multivariable Cox regression analysis, the 6-gene signature was indeed a significant prognostic factor (*p*-value < 0.001) while controlling for clinical covariates.

**Conclusions:**

Gene screening as a fast dimension reduction technique plays an important role in analyzing high-throughput data. The main contribution of this paper is to introduce a fundamental yet pragmatic model-free gene screening approach that aids statistical analysis of right-censored cancer data, and provide a lateral comparison with other available methods in the context of LUSC.

## 1. Introduction

Lung cancer is the leading cause of cancer-related death worldwide, with an estimated 1.8 million [1] deaths (from GLOBOCAN 2020 estimates). Among these, the non-small cell lung cancer (NSCLC) is the most common histological cell type and often presents in an advanced stage [2]. NSCLC is classified into adenocarcinoma, and squamous cell carcinoma (LUSC) subtypes, where LUSC comprises approximately 30% of all lung cancers [3]. Although molecular targeted therapy has been developed to significantly improve patient survival [4], tumour heterogeneity have been found to render the therapy ineffective [5]. For many patients the therapeutic options are still limited, especially for the LUSC subtype. The identification of biomarkers that contribute to early detection and effective treatment of LUSC is a vital yet ongoing research task, which is characterized by high-throughput data generated in a massive and fast manner by ‘omics’ technologies, such as transcriptomics, metabolomics and proteomics. As it is commonly believed that only a small portion of the clinical and genetic features are related to a certain endpoint of interest, a key aspect of the related statistical analysis is to extract core information by identifying low-dimensional sparse presentations of the predictive features, which is like finding a needle in a haystack for high-dimensional data. Traditional modeling techniques are handicapped, if the dimension (the number of variables) exceeds sample size. For example, the proportional hazards (PH) model has been widely used for predicting time-to-event outcomes, but the partial likelihood estimation is not appropriate for studying the simultaneous relationship of the high-throughput microarray data with the outcomes [6]. Hence, variable selection [7] becomes an indispensable part of the statistical analysis pipeline. However, when the number of variables is much larger than sample size, exact variable selection is often beyond the hope to achieve. Univariate analysis is commonly used to select significant biomarkers for downstream analysis without multiple testing correction [8–10], which accumulates false discoveries [11]. For large-scale multiple testing, the power to reject a non-null hypothesis while controlling for the family wise error rate through, for example, the Bonferroni adjustment, is greatly reduced as the number of tests increases [12]. Regularization methods such as LASSO [13] have also been applied to conduct gene selection for LUSC [14], but they can suffer from both statistical and computational issues if the number of features far exceeds sample size [15].

Recent years have seen rising attention to variable screening as a less ambitious yet efficient way to reduce dimension for ultrahigh dimensional data. Variable screening was first [15] introduced for linear model to quickly filter out redundant features through marginal independence learning based on the Pearson correlation. In other words, features are ranked based on their marginal associations with the outcome variable and unimportant genes are removed from the bottom of an ordered list. The screening mechanism asymptotically almost surely identifies all important predictors, and thus is called ‘sure independence screening’ (SIS). Since conjecturing about underlying model structure is presumably challenging in high dimensional spaces, more flexible approaches have emerged to avoid model specifications [15–19]. Screening has found applications ranging from quality control in the data processing step for genetic studies [20] to identifying predictive biomarkers for understanding biological mechanisms [21]. Notwithstanding the vast literature in feature screening for fully observed outcomes, the development of screening procedures to accommodate censoring has been less fruitful. Model-based methods include SIS for Cox PH model [22], the principled Cox sure screening [23] and the feature aberration at survival times screening [24], among others. In particular, SIS for Cox PH model [22] has been employed to discover prognostic gene signatures in breast cancer [25,26] and lung cancer [27,28]. However, if the association between the biomarkers and the survival outcomes cannot be well captured by the Cox model, which is rather difficult to check in practice for high dimensional data (due to the somewhat restrictive PH assumptions), SIS may fail to detect significant markers. Therefore, we argue that screening procedures that requires no model specification should be promoted, when there is insufficient information about data distribution and the underlying model structure. Existing model-free approaches include the quantile adaptive SIS [29], the censored rank independence screening (CRIS [30]), the survival impact index screening [31], the integrated powered density screening (IPO D [32]), and the robust screening via distance correlation [33]. Although the effectiveness of these aforementioned methods have been established through simulation studies [34], they have not been examined in the context of gene screening for cancer survival data. As appealing as the idea of variable screening is, the lack of application and dissemination hinders practical usage, which can benefit researchers from a wide biomedical domain.

In this paper, we proposed a model-free gene screening procedure for high-throughput right-censored cancer data based on the expected conditional characteristic function-based independence criterion (ECCFIC [35]). The ECCFIC correlation can be viewed as a nonlinear generalization of the classical coefficient of determination *R*^2^ since it requires no linearity or distributional assumptions and therefore can be used to achieve model-free screening. We applied the screening procedure to the TCGA LUSC dataset and identified a novel 6-gene signature for prognosis of LUSC patients. The performance of the screening procedure was evaluated via comparing and constrasting to existing alternatives.

## 2. Materials and Methods

### 2.1. Data Description

Gene expression data and clinical data for patients with LUSC were acquired from TCGA (https://cancergenome.nih.gov/ accessed on 30 September 2022) for model training and testing. In addition, information obtained from the Gene Expression Omnibus (GEO) database (GSE37745 [36] and GSE30219 [37]; https://www.ncbi.nlm.nih.gov/geo/(accessed on 30 September 2022)) was used for external validation. For a patient without an event (death), the overall survival time from first diagnosis was censored by the last follow-up date. Disease-free survival is defined as time to new tumor event after the initial treatment. Aside from 17,557 common genes in all datasets, 5 clinical covariates were also included in the analysis: age at diagnosis, gender, smoking history, metastasis and tumor stage. In total, 473 and 127 cases with completed data were extracted from TCGA and GEO datasets, respectively; 760 genes were excluded due to complete missing or low expression (with an interquartile range of 0). Table 1 summarizes the clinical and pathological characteristics of the TCGA patients. The majority of the patients were older than 60 at first diagnosis (82.6%) and had smoking history within 15 years (78.0%). Additionally, 81.6% of the patients had stage I and II squamous cell carcinoma, with only 1.5% of the patients presenting with stage IV carcinoma. 207 patients died during follow-up. The survival times range from 0.03 to 173.69 months, with a median of 21.19 months. The recurrence rate was 34.1%.

### 2.2. Sure Independence Screening for Right-Censored Data

We first introduce the concept of sure independence screening. Let *T* denote the time to event with respect to a certain cancer type, *C* denote the censoring time, *Y* ≔ min(*T*, *C*) denote the observed time and *δ* ≔ *I*(*T* ≤ *C*) denote the failure indicator, where *I*(·) is the indicator function. Let $X\in {\mathbb{R}}^{p}$ be the vector of all genes. Throughout the paper, we assume independent censoring, that is, (*T*, **X**) ⫫ *C*. Let A denote the index set of the influential genes, that is,

$$𝒜≔\left\{1\leq j\leq p:P(T>t|X)\text{ functionally depends on }X_{j}\right\}.$$

Our goal is to achieve gene screening, that is, to find a reduced index set that covers 𝒜 with cardinality smaller than *n*. Note that gene screening is less ambitious than exact gene selection that recovers 𝒜 precisely, but employed to quickly eliminate the majority of irrelevant genes and reduce the high dimensional data to a manageable subset.

### 2.3. The Screening Index

Before we introduce the procedure of gene screening, we briefly review the measure that will be used to assess the dependence between the survival time and each candidate gene. Let *U* and *V* be two random variables. The generalized ECCFIC [35] for testing *U* ⫫ *V* is defined as

$${ℋ}_{K}^{2}(U|V)≔E_{V}E_{U\left|V,U^{\prime }\right|V}K\left(U,U^{\prime }\right)-E_{U,U^{\prime }}K\left(U,U^{\prime }\right),$$

for a characteristic [38] positive definite kernel K:ℝ×ℝ→ℝ, where *E*_*U*|*v*,*U*′|*v*_ denotes *E*(·|*V* = *v*, *V*′ = *v*) and (*U*′, *V*′) is an independent and identically distributed copy of (*U*, *V*). Examples of characteristic kernels include Gaussian, Laplacian, inverse multiquadratics, and distance-induced kernels [39]. A corresponding correlation measure is then defined as

$$\rho_{K}(U|V)≔\frac{{ℋ}_{K}^{2}(U|V)}{{ℋ}_{K}^{2}(U|U)},$$

where ${ℋ}_{K}^{2}(U|U)=E_{U}K(U,U)-E_{U,U^{\prime }}K\left(U,U^{\prime }\right)$. It can be showed that 0 ≤ *ρ*_*K*_(*U*|*V*) ≤ 1, where *ρ*_*K*_(*U*|*V*) = 0 if and only if *U* and *V* are independent and *ρ*_*K*_(*U*|*V*) = 1 if and only if *U* is a function of *V*. The ECCFIC correlation can capture nonlinear dependence with the kernel trick and thus, is more general than the coefficient of determination or the Pearson correlation coefficient.

The Nadaraya-Watson estimator of ${ℋ}_{K}^{2}(U|V)$ relying on a selected smoothing kernel G:ℝ→ℝ and a tuning bandwidth h≔h(n)∈ℝ is given by

$${ℋ}_{K,G,n}^{2}(U|V)≔\frac{1}{n^{5}}\sum_{t_{1},t_{2},t_{3},t_{4},t_{5}=1}^{n}\frac{G_{t_{1}t_{2}}G_{t_{1}t_{3}}d_{t_{2}t_{3}t_{4}t_{5}}}{\frac{1}{n^{2}}\sum_{s_{1},s_{2}=1}^{n}G_{t_{1}s_{1}}G_{t_{1}s_{2}}},$$

where *Gts* ≔ *G*_*h*_(*V*_*t*_ − *V*_*s*_), *G*_*h*_(*v*) ≔ *h*^−*q*^*G*(*v*/*h*), $d_{t_{2}t_{3}t_{4}t_{5}}≔K_{t_{2}t_{3}}-K_{t_{2}t_{4}}-K_{t_{3}t_{5}}+K_{t_{4}t_{5}}$ and *K*_*ts*_ ≔ *K*(*U*_*t*_, *U*_*s*_). Furthermore, a natural estimator of ${ℋ}_{K}^{2}(U|U)$ is given by

$${ℋ}_{K,n}^{2}(U|U)≔\frac{1}{n}\sum_{i=1}^{n}K\left(U_{i},U_{i}\right)-\frac{1}{n^{2}}\sum_{i_{1},i_{2}=1}^{n}K\left(U_{i_{1}},U_{i_{2}}\right).$$

Then the ECCFIC correlation can be estimated by

$$\rho_{K,G,n}(U|V)≔\frac{{ℋ}_{K,G,n}^{2}(U|V)}{{ℋ}_{K,n}^{2}(U|U)}.$$

In practice, the bandwidth *h* is often set to $1.06\tilde{\sigma }n^{-1/5}$, where $\tilde{\sigma }$ is estimated by the sample standard deviation of V [40].

### 2.4. The Sreening Algorithm

We now provide an algorithm to achieve gene screening for high-throughput rightcensored cancer data. The ECCFIC correlation between *U*_*T*_ ≔ *F*_*T*_(*T*) and $U_{X_{j}}≔F_{X_{j}}\left(X_{j}\right)$ is adopted to quantify the importance of the individual gene *X*_*j*_ (*j* = 1, …, *p*), where *F*_*T*_(·) is the cumulative distribution function (CDF) of *T* and $F_{X_{j}}(\cdot )$ is the CDF of *X*_*j*_. Note that *T* ⫫ *X*_*j*_ if and only if $U_{T}⫫U_{X_{j}}$, but we choose to work with the later condition, since (1) *T* is not observable but *U*_*T*_ can be easily estimated by the well-known Kaplan–Meier estimator, and (2) $U_{X_{j}}{}^{\prime }\text{s}$ provide robustness to heavy tails or outliers of the gene expression.

For a characteristic kernel *K* of choice, let $w_{j}≔\rho_{K}\left(U_{T}|U_{X_{j}}\right)$. Given the observed data ${\left\{X_{i},Y_{i},\delta_{i}\right\}}_{i=1}^{n}$, the steps of our algorithm are as follows:

1. Estimate the survival function by the Kaplan–Meier estimator as
$${\hat{F}}_{T}(t)≔1-\prod_{i=1}^{n}{\left(1-\frac{1}{\sum_{l=1}^{n}I\left\{Y_{l}\geq Y_{i}\right\}}\right)}^{\delta_{i}I\left\{Y_{i}\leq t\right\}}$$
  and compute the empirical CDF of *X*_*j*_ as ${\hat{F}}_{X_{j}}(x)=\frac{1}{n}\sum_{i=1}^{n}I\left\{X_{ij}\leq x\right\}$;
2. Treat ${\left\{{\hat{F}}_{X_{j}}\left(X_{ij}\right),{\hat{F}}_{T}\left(Y_{i}\right)\right\}}_{i=1}^{n}$ as the observed data of $\left(U_{X_{j}},U_{T}\right)$ and compute the sample correlation ${\hat{w}}_{j}≔\rho_{K,G,n}\left(U_{T}|U_{X_{j}}\right)$ for *j* = 1, …, *p*.
3. Let $\hat{𝒜}≔\left\{1\leq j\leq p:{\hat{w}}_{j}\text{ is among the first }d\text{ largest of all }\right\}$.

**Figure 1.**
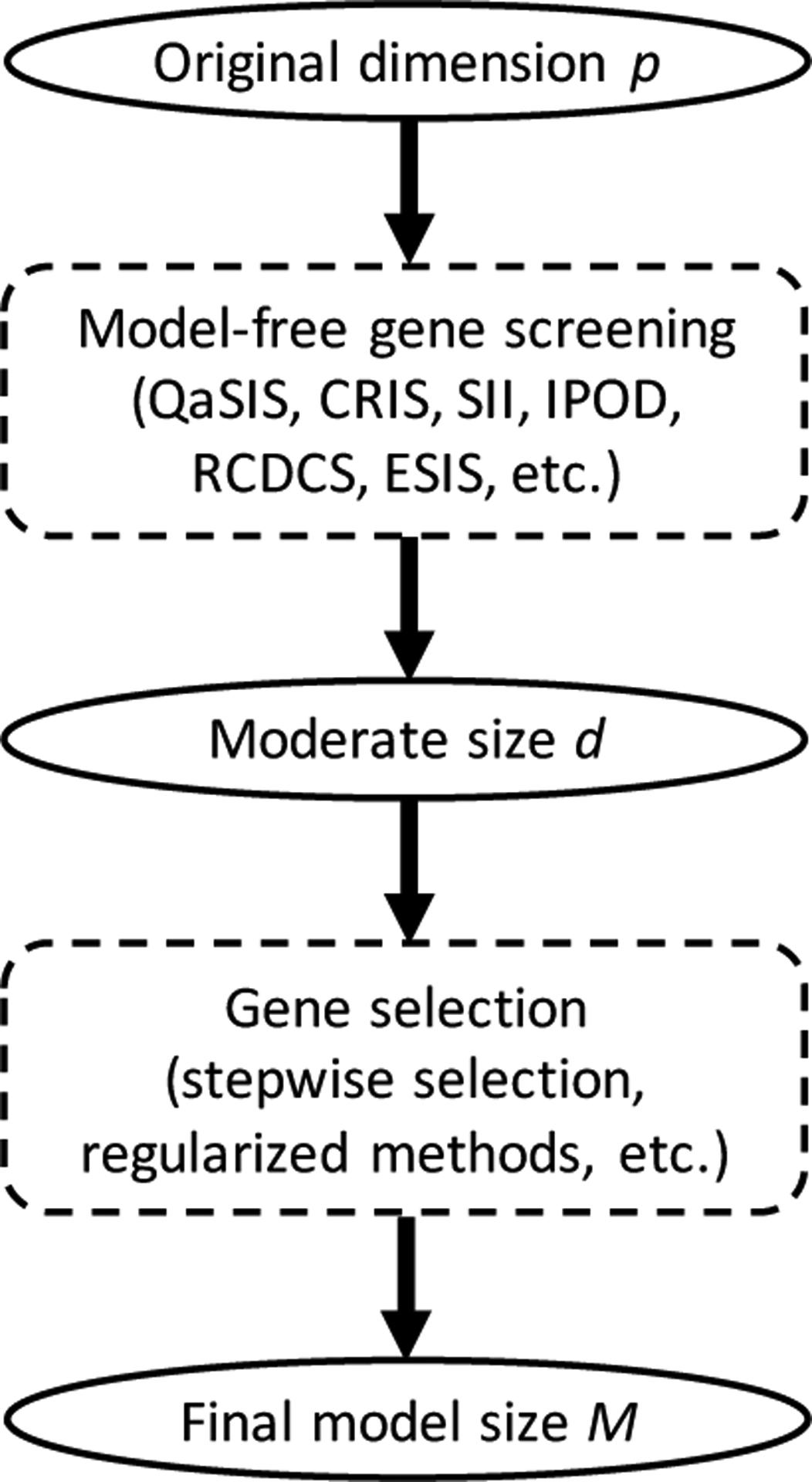
Overall diagram of gene screening and selection procedures.

We henceforth refer to our procedure as the ECCFIC-based sure independence screening, or ESIS for short. In practice, common choices of *d* are [*n*/log(*n*)], 2[*n*/log(*n*)], 3[*n*/log(*n*)], and *n* − 1 [15,16]. Once the dataset is sufficiently downsized by ESIS, traditional lower dimensional methods can be used afterwards for gene selection and statistical inference (Figure 1). It is noteworthy to point out that ESIS does not impose any model assumptions on the distribution of *T*|**X**. The R code to implement the proposed algorithm is available at https://github.com/cke23/GeneScreeningDemo1 (accessed on 30 September 2022).

### 2.5. Application

The TCGA data were divided into a training set and a testing set in a ratio of 4:1 by stratified randomization based on censoring. The training set was comprised of 379 samples and the testing set was comprised of 94 samples. We first performed ESIS on the training set and pre-selected 379 − 1 = 378 genes. The characteristic kernel as well as the smoothing kernel were both chosen to be the Gaussian kernel. A Penalized Cox model with LASSO regularization (abbreviated as PenCox henceforth) was then applied to the reduced training data for further gene selection and prognosis simultaneously via R package glmnet. The optimal tuning parameter was determined through 10-fold cross validation. A patient’s risk score was calculated as the linear predictor of the fitted PenCox model. Patients were classified as having a high-risk gene signature or a low-risk gene signature, with the median risk score of the training group being the cutoff. The same cutoff value was also applied when assigning the test samples (TCGA LUSC data) and the external validation samples (GEO datasets) into two risk groups. To evaluate the predictive performance of the PenCox model built upon the ESIS-selected genes, the Kaplan–Meier curves of the two risk groups for both overall survival and disease-free survival were compared using the log-rank tests. Moreover, the time-dependent receiver operating characteristic (ROC) curve along with the area under the curve (AUC) were calculated. Finally, a Cox model was fitted to the entire TCGA dataset to make inference about independent prognostic factors associated with survival, and the selected gene signature, age, gender, tumor stage, and smoking history were used as covariates. The same analysis preceded by two existing screening methods, namely CRIS [30] and IPOD [32], were also conducted, respectively, for comparisons. As a baseline model, we performed a naive screening procedure followed by PenCox. That is, we ranked the genes by their variations and select the top 700 for downstream analysis [41]. The purpose of the baseline model was to evaluate the classical regularization method with relatively high dimensional data and the naive screening procedure assisted to reduce the computational cost. In total, four models were included for comparisons: Naive+PenCox, CRIS+PenCox, IPOD+PenCox, and ESIS+PenCox.

## 3. Results

**Figure 2.**
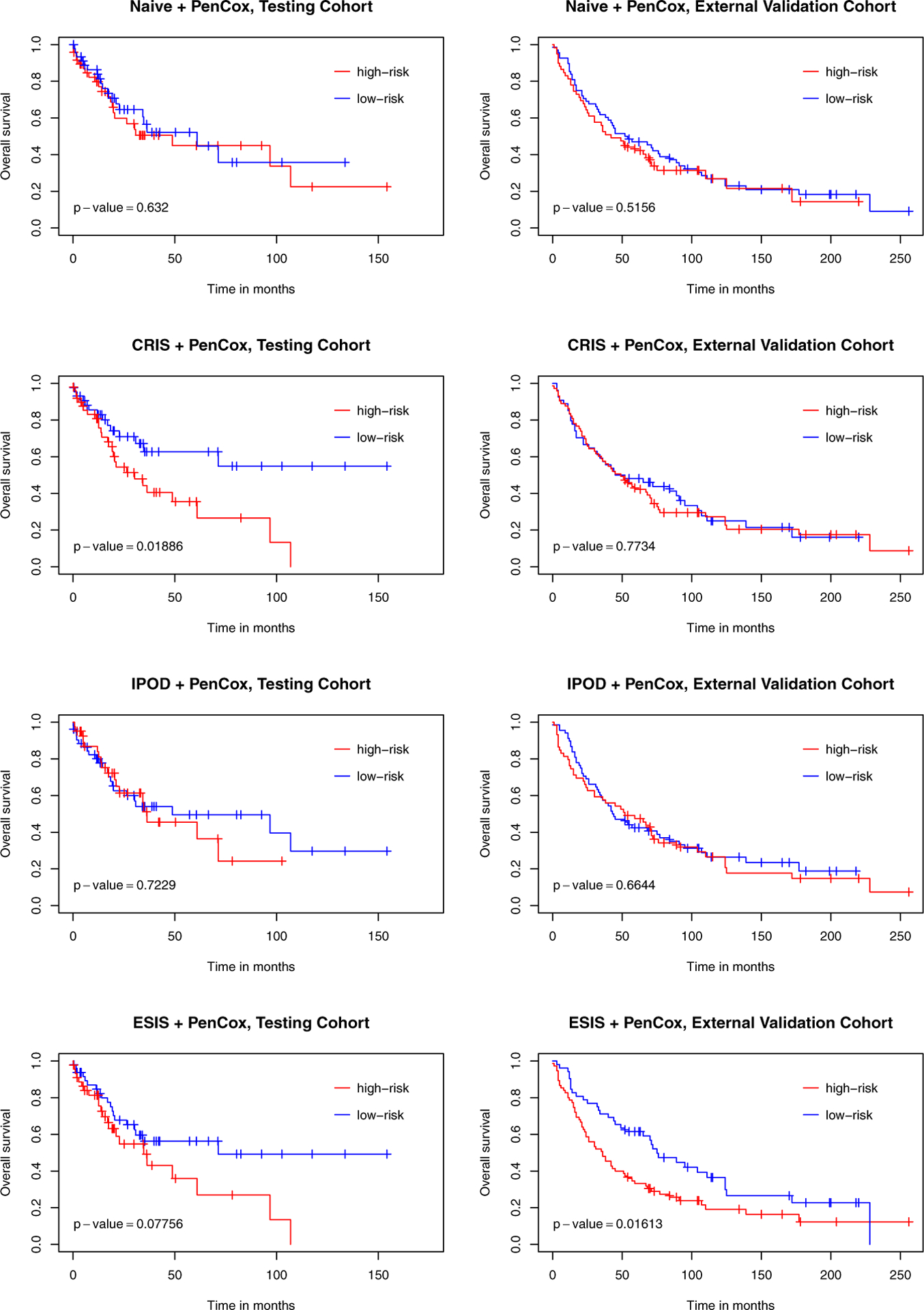
Kaplan–Meier curves of overall survival for test (TCGA) and validation (GEO) cohorts varying with the risk level determined by the four competing models. *p*-values were obtained from the log-rank tests contrasting the two risk groups.

**Figure 3.**
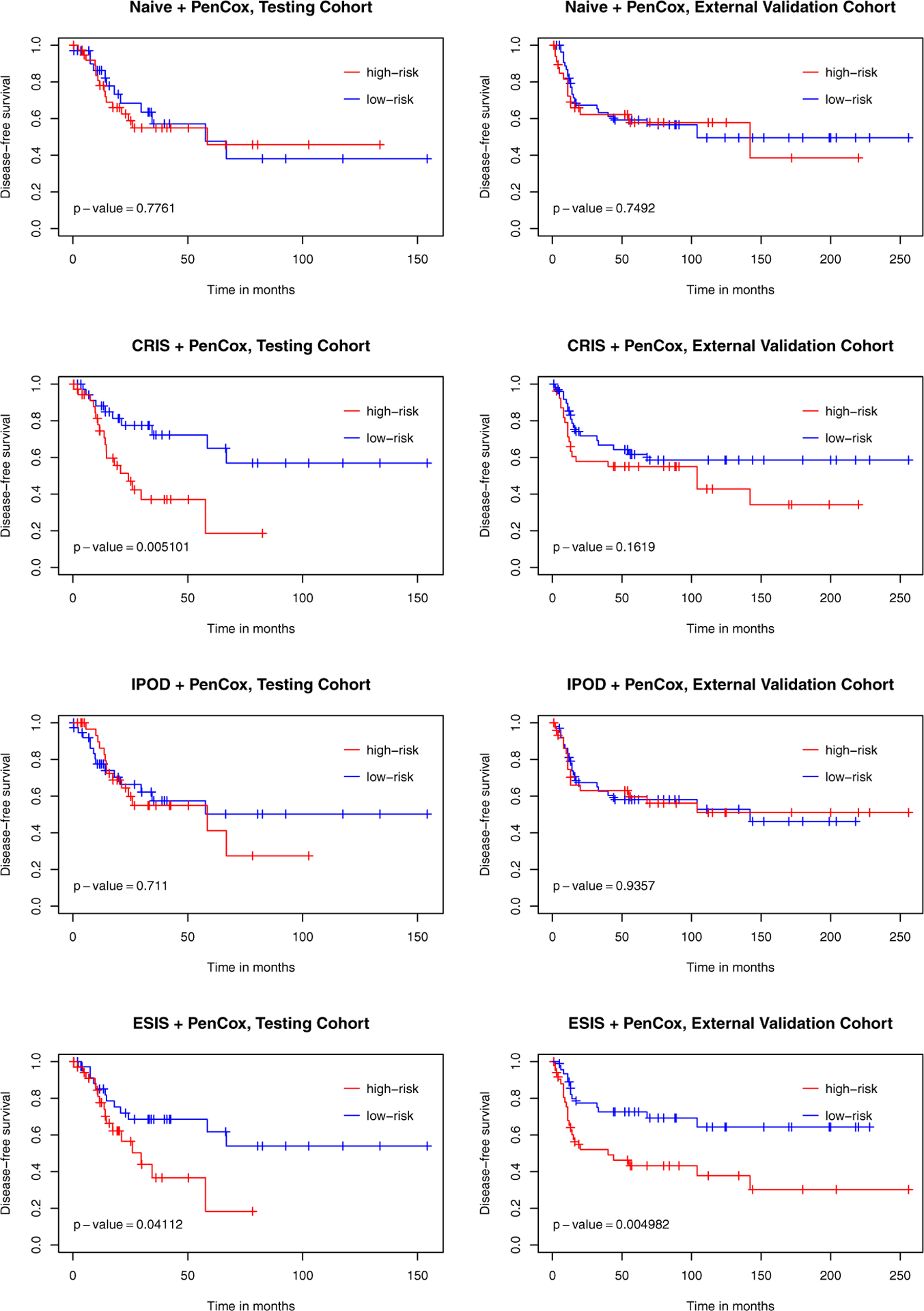
Kaplan–Meier curves of disease-free survival for test (TCGA) and validation (GEO) cohorts varying with the risk level determined by the four competing models. *p*-values were obtained from the log-rank tests contrasting the two risk groups.

**Figure 4.**
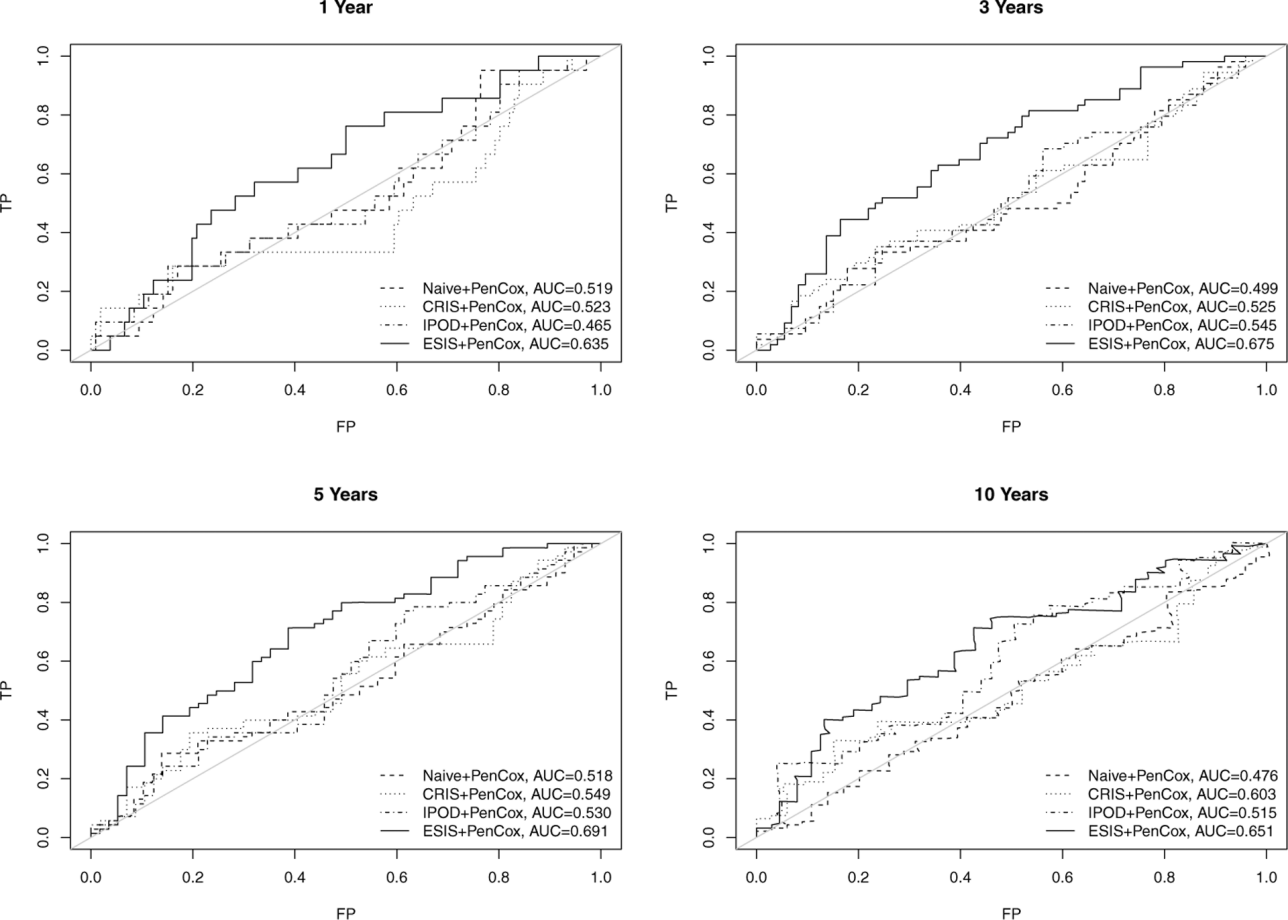
ROC curves of overall survival predicted by the four competing models on the external validation data.

Table 2 lists the influential genes selected by each of the four competing models. All models successfully distinguished the two risk groups for the training data with *p*-values < 0.001. For the testing samples, the ESIS+PenCox model also led to a separation between the two groups (*p*-value = 0.078). Patients with a high-risk gene signature had a shorter median overall survival than those with a low-risk gene signature (34.7 months vs. 71.3 months). Moreover, patients with a high-risk gene signature were associated with a shorter disease-free survival than patients with a low-risk gene signature (29.7 months vs. not reached for median survival, *p*-value = 0.041). The same observation held for the subgroup of patients with metastasis (34.4 months vs. not reached for median disease-free survival; *p*-value = 0.010). For the external validation samples, the ESIS+PenCox provided the best stratification among the four models in terms of overall survival (*p*-value = 0.016) and disease-free survival (*p*-value = 0.005 for all patients and *p*-value = 0.083 for patients with metastasis). The prognostic indices based on the genes selected by the other screening methods were less informative, leading to insignificant discrepancies between the two risks groups in the validation data. The PenCox model with naive screening suffered from the high dimensionality (700 genes) and failed to predict overall survival and disease-free survival effectively. Figure 2 shows the overall survival curves for high-and-low risk groups in the testing and external validation cohorts, while results for disease-free survival are presented in Figure 3. Figure 4 displays the ROC curves at 1, 3, 5 and 10 years for the competing models on the external validation data. The results also suggest that the ESIS+PenCox model provided the best predictions.

From the multivariable Cox regression (Table 3), the 6-gene signature selected by ESIS+PenCox was a strong predictor with an hazard ratio of 12.59 (*p*-value < 0.001), adjusted for other clinical covariates. There was a 2% increase in the expected hazard relative to a one year increase in age (*p*-value = 0.008). Subjects with first or the second stage of cancer experienced reduction of hazard by 41% (*p*-value = 0.003) and 37% (*p*-value = 0.018), respectively, compared to those in later stages. Current smokers were associated with worse prognosis (*p*-value = 0.005).

Finally, we highlight some biological insights associated with the genes selected by ESIS+PenCox. The protective gene SDHAF3 has been found to be involved in the maturation of succinate dehydrogenase (SDH) genes, which are known as classical tumor suppressors [42]. Following the suppression of SDH genes, an accumulation of succinate results in stabilizing HIF-*α*, thereby promoting angiogenesis and ROS production [43]. In particular, inhibition of SDHB induces the transition to anaerobic metabolism, better known as the Warburg effect, which is widely observed in human cancers [44]. Single nucleotide polymorphisms (SNPs) in SDH genes have been associated with the clinical outcome of NSCLC patients [45]. Studies have shown that the indication of IBTK may be expanded beyond hematological malignancies [46]. In several cancers, IBTK functions to sustain tumorigenesis and cell survival [47]. For instance, IBTK has been identified as a risk gene of NSCLC, owing to its association with KRAS, AKT1, BRAF and MAPK1 [48]. It has also been revealed that FAM65A binds to Rho GTPases that regulate cancer cell migration [49,50]. FAM65A is a component of the gene expression profiles for atopy [51] and pulmonary function impairment [52]. Mon1 mediates the transition from early-to-late endosome in metazoa by switching Rab5 for Rab7 via guanine nucleotide exchange factors [53]. Mon1b, the mammalian homolog of Mon1, interacts with Numb for docking of early endosomes [54]. Mon1b is elevated in colon cancer, with its knockdown in vitro leading to a reduction of proliferation, migration, and invasion [55]. There is evidence that NACC2/RBB inhibits cell cycle progression and promotes apoptosis by enhancing the p53 pathway [56]. NACC2 has also been identified as an NTRK fusion protein, specifically in pilocytic astrocytoma [57,58]. NTRK gene fusions lead to constitutive activation of TRK kinases in multiple cancers, thereby making them promising candidates for chemotherapeutic drug development [59]. The protective gene LOC641845/STMP1 is a short trans-membrane mitochondrial protein that participates in the regulation of cellular respiration [60]. Although this gene has not been widely studied, it appears to have a role in Paget’s disease of the bone [61].

Thanks to the ENCODE transcription factor target datasets [62,63] that are available on the Harmonizome database [64], we identified two transcription factors, E2F4 and ELF1, which regulate five out of the six genes selected by the ESIS+PenCox model: NACC2, FAM65A, MON1B, IBTK, and SDHAF3. E2F4 is a member of the E2F family of transcription factors which regulate the expression of key genes implicated in cell division [65]. In particular, E2F4 belongs to a subclass of repressive E2Fs that play a role in cell cycle exit and terminal differentiation [65]. ELF1 belongs to the E26 transformation specific (ETS) family of transcription factors which regulate the expression of genes involved in several processes that are considered the hallmarks of cancer [66,67]. ELF1 binds to the HER2 promoter and is upregulated in several cancers (prostate, ovarian, breast, leukemia, lymphoma) [66].

## 4. Discussion

Finding prognostic gene signatures for cancer survival is a vital task in biomedical research. Since it is commonly believed that only a small portion of genes are related to a certain outcome, how to recover the most influential subset from massive data becomes a challenge in related statistical analysis. Traditional variable selection methods such as stepwise selection can only be applied when the number of variables is smaller than the sample size. Researchers often use prior knowledge or univariate analysis to select genes for downstream analysis, which lacks quantitative justification and could hinder the discovery of novel gene markers. Although regularization methods have also been widely used, they can be unstable for high-throughput data (the number of genes far exceeds the number of samples). Fast and effective variable screening tools for high dimensional survival data have been emerging in the past decade in the statistics literature. However, the dissemination of these attractive methods to biomedical fields is limited. In this paper, we proposed a novel sure independence screening procedure for identifying prognostic genes in LUSC. Our approach was able to reduce the dimension efficiently while preserving influential genes that lead to biologically sensible findings and provide better prognosis for LUSC in comparison with competing methods. The proposed gene screening tool is fundamental and general, and thus can be readily applicable to other cancer databases with right censored survival. Classical gene selection and prognostic modeling can be conducted subsequently after the dataset is downsized through screening.

Admittedly, this paper poses some open questions besides what it solves. Our method allows a variety of kernels for detecting important genes involved with different types of model structure, but kernel selection is commonly challenging and requires a large amount of practical experience for the researchers. As future work, we plan to develop a composite algorithm integrating results of distinct kernels. Besides, in many applications, researchers know from previous investigations that certain features are responsible for the survival outcomes or should be controlled for in the studies. Examples include TNM clinical stage, pathological stage, metastasis, age, gender, smoking history, and known gene markers. Although some of the covariates were included in the final prognostic modeling stage in our study, they may also assist in the selection of important genes while being shielded in the screening procedure. In future work, we also plan to investigate such conditional screening procedures that can incorporate prior information to improve the screening power.

## 5. Conclusions

We developed a novel and powerful model-free gene screening approach that aids statistical analysis of high-throughput right-censored data. The application to TCGA LUSC data provided a paradigm of its implementation combining classical gene selection and prognostic modeling. As a result, we discovered a novel and effective six-gene model to predict the prognosis of patients with LUSC. It is expected that this presented work will be a desired addition to a cancer epidemiologist’s toolbox.

## Figures and Tables

**Table 1.** Summary of clinical and pathological characteristics.

Variables	Frequency (Percent)
Age	
Less than 50	15 (3.2%)
50–59	67 (14.2%)
60–69	178 (37.6%)
70–79	186 (39.3%)
80 or greater	27 (5.7%)
Gender	
Female	125 (26.4%)
Male	348 (73.6%)
Smoking History	
Current reformed smoker for ≤ 15 years	236 (49.9%)
Current reformed smoker for > 15 years	81 (17.1%)
Current reformed smoker, duration not specified	5 (1.1%)
Current smoker	133 (28.1%)
Lifelong non-smoker	18 (3.8%)
Lymph Node Metastasis	
N0	302 (63.8%)
N1, N2, N3	165 (34.9%)
NX	6 (1.3%)
Distant Metastasis	
M0	386 (81.6%)
M1, M1a, M1b	7 (1.5%)
MX	80 (16.9%)
Pathological Stage	
I	236 (49.9%)
II	150 (31.7%)
III	80 (16.9%)
IV	7 (1.5%)

**Table 2.** Genes selected by the four competing models. A risk gene with a positive coefficient from the fitted PenCox model is denoted by “+”, while a protective gene with a negative coefficient is denoted by “−”.

Model (No. of Genes Selected)	Gene Names
Naive + PenCox (6)	PCDHA5(+), C9ORF131(+), PM20D1(+), PCDHA3(+), FAM196B(+), PITX3(−)
CRIS + PenCox (10)	CCDC79(+), LCN1(+), GPR78(+), SSX1(+), CCKAR(+), SLC10A2(+), STARD6(−), GUCY2F(−), DPPA2(+), LINC00628(+)
IPOD + PenCox (4)	TRIM58(+), C9ORF131(+), PKNOX2(+), PCDHGA11(+)
ESIS + PenCox (6)	NACC2(+), FAM65A(+), LOC641845(−), MON1B(+), IBTK(+), SDHAF3(−)

**Table 3.** Multivariable Cox regression analysis of the risk of death against the 6-gene signature identified by ESIS+PenCox and other clinical covariates. CI denotes confidence interval.

Variable	Hazard Ratio (95% CI)	*p*-Value
6-gene signature	12.59 (4.11, 38.56)	<0.001
Age	1.02 (1.01, 1.04)	0.008
Gender		
Male	0.92 (0.67, 1.28)	0.629
Female	-	-
Tumor stage		
I	0.59 (0.42, 0.83)	0.003
II	0.63 (0.43, 0.92)	0.018
III or IV	-	-
Smoking history		
Lifelong non-smoker	1.94 (0.83, 4.54)	0.126
Current smoker	1.54 (1.14, 2.07)	0.005
Current reformed smoker	-	-

## Data Availability

The LUSC data used in this paper is available at the TCGA repository https://cancergenome.nih.gov/ (accessed on 30 September 2022) and the GEO database https://www.ncbi.nlm.nih.gov/geo/ (accessed on 30 September 2022). The R code for the proposed algorithm is available at https://github.com/cke23/GeneScreeningDemo1 (accessed on 30 September 2022).
